# Insight and Development of Advanced Recombinant Adeno-Associated Virus Analysis Tools Exploiting Single-Particle Quantification by Multidimensional Droplet Digital PCR

**DOI:** 10.1089/hum.2021.182

**Published:** 2022-09-16

**Authors:** Jeanette Zanker, Sara Lázaro-Petri, Daniela Hüser, Regine Heilbronn, Adrien Savy

**Affiliations:** Department of Neurology, AG Gene Therapy, Berlin Institute of Health, Charité—Universitätsmedizin Berlin, Corporate Member of Freie Universität Berlin, Humboldt-Univerisität zu Berlin, Berlin, Germany.

**Keywords:** gene therapy, AAV, ddPCR, genome integrity, bias, quality control

## Abstract

Recombinant adeno-associated virus (rAAV) has become the most widely used vector in the gene therapy field with hundreds of clinical trials ongoing and already several products on the market. AAV's physicochemical stability, and the various natural and engineered serotypes allow for targeting a broad range of cell types and tissue by diverse routes of administration. Progressing from early clinical studies to eventual market approval, many critical quality attributes have to be defined and reproducibly quantified, such as AAV stability, purity, aggregates, empty/full particles ratio, and rAAV genome titration. Droplet digital PCR (ddPCR) is becoming the tool of choice to perform absolute quantification of rAAV genomes. In the present study, we have identified critical parameters that could impact AAV titration and characterization accuracy, such as Poisson distribution confidence interval, primers/probe position, and potential aggregates. Our work presents how ddPCR can help to better characterize AAV vectors on the single particle level and highlights challenges that we are facing today in terms of AAV titration.

## INTRODUCTION

Adeno-associated virus (AAV) is a nonenveloped virus, which belongs to the *parvoviridae* family. Its single-stranded genome is flanked by hairpin-shaped inverted terminal repeats (ITRs) on both ends. Recombinant AAVs (rAAV) are widely used in the gene therapy field, from preclinical research to market approval with already two rAAV-based products approved by the Food and Drug Administration (FDA) (Luxturna^®^—rAAV2-RPE65 for treatment of inherited retinal disease, and Zolgensma^®^—rAAV9-SMA for spinal muscular atrophy).

rAAV production consists of transfection of plasmids, one with the gene of interest (GOI) embedded between the two ITRs, and one or two containing AAV *rep* and *cap* genes and helper virus functions in trans.^1^ rAAV genomes differ by their promoter, GOI, introns, and/or polyA sequences. The vast majority of rAAV vectors use ITRs from AAV serotype 2^1–3^ representing universal DNA templates for rAAV genome quantification by quantitative PCR (qPCR).^4^ However, it has been described that this ITR titration method could lead to an overestimation of the rAAV titer due to the variant ITR structure in the plasmid backbone used as template for the standard curve. This has since been overcome by using free ITR during the qPCR experiments.^5^

Traditional qPCR-based rAAV genome titration methods have already been extensively described.^5–9^ Even though being widely used and accepted, qPCR may lead to various biases coming from the plasmid standard curve quality (quantification of linearized vs. supercoiled DNA), general PCR efficiency, and/or AAV genome extraction method.^7^ Two reference standard materials (AAVRSM2 and AAVRSM8) can be purchased from ATCC (ATCC VR-1616^®^ and ATCC VR-1816^®^). Availability of these two Reference Standard Materials (RSM) allows for direct method comparison and thus reduces the risk of titration variations between laboratories.

However, as described previously,^10,11^ variations within the 95% confidence limit accepted range, despite following the same standard operation procedure, may still result in substantial differences (AAVRSM2 2.70 × 10^10^ vector genome per mL [vg/mL] to 4.75 × 10^10^ vg/mL and for the AAVRSM8 3.05 × 10^11^ vg/mL to 1.09 × 10^12^ vg/mL).

Variations may be even higher if each laboratory uses variant equipment and proprietary protocols. Different qPCR techniques (TaqMan^®^ or SYBR), primers/(probes), standard curve generation, analysis, and/or rAAV genome extraction methods could directly impact titration accuracy. Unfortunately, standard curves are not available as reference standard material.

Droplet digital PCR (ddPCR), by its mode of action avoids the need for a standard curve since concentrations are calculated based on the Poisson distribution. Thus, by obviating one of the potential biases of inaccurate AAV titration, ddPCR has already been described for quantification and characterization of rAAV genomes, either after genome extraction or by using intact viruses to generate droplets.^12–15^ In the present study, we describe how the sample itself, its pretreatment, and the primers/probes localization can impair the accuracy of AAV titration by ddPCR for the AAV reference standard material (AAVRSM8) and for our own rAAV1 batches.

## MATERIALS AND METHODS

### Cell culture

HEK-293 (ATCC—no. CRL-1573) were cultivated in Dulbecco's modified Eagle's medium (DMEM) (Gibco—no. 41966-029) supplemented with 10% fetal bovine serum (FBS) (Pan Biotech—no. P30-3031) and were maintained in a 37°C humidified incubator at 5% CO_2_.

### Plasmid production

pTR-UF11 was transformed into electrocompetent *Escherichia coli* surE bacterial strain. Briefly, bacteria were grown overnight at 37°C, 125 rpm (SM-30 orbital, Edmund Bühler GmbH, Germany) in 250 mL of LB + Ampicillin, and purified with the QIAGEN Plasmid Maxi Kit (QIAGEN—no. 12162) following the manufacturer's protocol. DNA pellet was resuspended in TE Buffer.

### AAV1 production

rAAV1 were produced by cotransfection of pTR-UF11^3^ and pDP1-rs (Plasmid Factory—no. PF401) with PEI (Polyethylenimine—PolyScience—no. 23966-1) in adherent HEK-293 with DMEM (Gibco—no. 41966-029) supplemented with 10% FBS (Pan Biotech—no. P30-3031). Briefly, 72 h post-transfection, adherent cells were detached by addition of 12.5 mM final concentration of ethylenediaminetetraacetic acid (EDTA) 0.5 M. Supernatant and cells were treated with 30 mM MgCl_2_, 0.5% Triton X-100 and 1 000 U (for 500 mL of bulk) of Benzonase (Roche—no. 1.01654-0001) for 3 h at 37°C under 140 rpm (orbital shaker—Heathrow Scientist).

Culture bulk was then clarified with a Pall Preflow filter (no. DFA3001UBC). rAAV1 were purified from the clarified product with Poros Go-Pure AAVX prepacked column (Thermo Fisher—no. A36652) and an Äkta purifier (Cytiva). rAAV1 were eluted with 100 mM Glycine buffer at pH 2.5 and neutralized with Tris pH 8.5. rAAV1 were concentrated with PBS supplemented with 625 mM KCl and 10 mM MgCl_2_ with an Amicon 100 kDa (Millipore—no. UFC910024).

### rAAV titration

rAAV1 titration is performed by ddPCR with QX200 (Bio-Rad). For native AAV without genome extraction, six times 1 μL of rAAV1 and 1 μL of AAVRSM8 (used as internal control) were treated with 1, 5, or 10 U of DNase I (Thermo Fisher—no. EN0521) for 30 min at 37°C in 17 μL of DNase I Buffer (Tris HCl 1 M, CaCl_2_ 0.1 M, MgCl_2_ 1 M) supplemented with 0.05% F-68 (Gibco—no. 24040-032). Two microliters of 50 mM EDTA were then added, and samples were heated at 65°C for 10 min. Samples were serially diluted up to 1:1,000,000 (for three of them) and 1:2,000,000 (for three of them), AAVRSM8 was diluted 1:1,000,000 times in ddH_2_O + 0.05% F-68.

AAV were quantified by ddPCR in ddPCR Supermix for Probes (no deoxyuridine triphosphate) (Bio-Rad —no. 1863025), with cytomegalovirus (CMV), bovine growth hormone (bGH), green fluorescent protein (GFP), and Beta-Lactamase primers/probes (Primer Table [either Eurofins or Integrated DNA Technologies; Table 1]), following the manufacturer's instructions.

**Table 1.** Primers/probes used in the current study, with the probe modifications

Primer Name	5′–3′ Sequence	Modifications
CMV forward	CCTATTGACGTCAATGACGG	
CMV reverse	GATGTACTGCCAAGTAGGAAAG	
CMV probe	ATGCCCAGTACATGACCTTATGGG	[5′]6-FAM—[3′]BHQ1
bGH forward	CTGTGCCTTCTAGTTGCCAG	
bGH reverse	GGAAAGGACAGTGGGAGTGG	
bGH probe	CCTTCCTTGACCCTGGAAGG	[5′]HEX—[3′]BHQ1
Beta-lac forward	CACAACATGGGGGATCATG	
Beta-lac reverse	GTCGTTTGGTATGGCTTCATTC	
Beta-lac probe	ACTCGCCTTGATCGTTGGG	[5′]HEX—[3′]BHQ1
GFP forward	GTCAAGTTCGAAGGTGACACC	
GFP reverse	GGCCGAGAATGTTTCCATCC	
GFP probe FAM	AGAATCGAGCTGAAGGGCATTGAC	[5′]6-FAM—ZEN—[3′]IBFQ
GFP probe HEX	AGAATCGAGCTGAAGGGCATTGAC	[5′]HEX—ZEN—[3′]IBFQ

bGH, bovine growth hormone; CMV, cytomegalovirus; FAM, fluorescein; GFP, green fluorescent protein; HEX, hexachloro fluorescein; IBFQ, Iowa Black FQ.

Droplets were generated with the QX200 Droplet Generator following the manufacturer's instructions (Bio-Rad). Droplets were read with the QX200 Droplet Reader and results were analyzed with QuantaSoft Analysis Pro Software (V1.0.596- Bio-Rad). Samples were analyzed only if more than 15,000 droplets were read.

rAAV1 titer is determined by the average of the six technical replicates, and validated if the coefficient of variation (CV) is below 20% and if the AAVRSM8 value is enclosed in the acceptance criteria defined by the RSM group.^11^

When indicated, rAAV genomes were extracted with High Pure Viral Nucleic Acid Extraction Kit (Roche—no. 11858874001) following the manufacturer's instructions and eluted in elution buffer supplemented with 0.05% F-68. Briefly, 5 μL of rAAV were digested with 10 U DNaseI for 30 min at 37°C in a final volume of 50 μL DNase I buffer (see above). DNase I was heat inactivated for 10 min at 65°C in combination with the addition of 2 μL of 50 mM EDTA. Fifty microliters of Proteinase K (10 mg/mL) were added and AAV capsids were digested for 15 min at 72°C. rAAV genomes were purified through high pure viral nucleic acid columns provided in the kit.

Thermal cycling conditions: (1) 95°C (10 min) Ramp 2.5°C/s; (2) 94°C (30 s) Ramp 2.5°C/s; (3) 60°C (1 min) Ramp 2.5°C/s (2–3 × 40); (4) 98°C (10 min). Reference standard material was obtained from ATCC: AAVRSM8 (VR-1816). AAVRSM8 was manufactured as described.^11^

### Graphical representation and statistical analysis

Graphical representation and statistical analysis were performed using Prism V9.02 GraphPad software.

### Electron microscopy

For the negative staining, carbon-coated mesh grids were hydrophilized with Alcian Blue (Sigma-Aldrich, St. Louis) solution (1% in 1% acetic acid in water) for 10 min, followed by washing steps on drops of distilled H_2_O. The grids were placed on 15 μL of particle solution for 10 min, followed by removing the solution by a filter paper, washing steps on drops of distilled H_2_O and finally placed on a drop of freshly prepared solution of 1% aqueous uranyl acetate (Serva, Heidelberg, Germany) for 20 s. Finally, the drop on the grid was sucked off with a filter paper and the grid dried in a grid box. The images were acquired on a Zeiss Leo 906 electron microscope (Carl Zeiss, Oberkochen, Germany) at 80 kV acceleration voltage equipped with a slow scan 2K CCD camera (TRS, Moorenweis, Germany).

### Alkaline gel

Two micrograms of pTR-UF11 were digested in 20 μL either with 0.1 U/μL of *Sma*I (New England Biolabs—no. RS0141S) or with 0.1 U/μL of *Msc*I (New England Biolabs—no. R0534L) following the manufacturer's instructions. A total of 4 × 10^11^ vector genomes of rAAV1 were treated in a final volume of 150 μL in DNAse Buffer (Tris HCl 1 M, CaCl_2_ 0.1 M, MgCl_2_ 1 M) with 0.15 U/μL of DNaseI (Thermo Fisher—no. EN0521) at 37°C for 30 min, and inactivated with 5 mM EDTA at 65°C for 10 min.

Capsids were digested with 5 mg/mL of Proteinase K (Roth—no. 7528.5) at 70°C for 20 min. DNA from rAAV1 genomes and digested plasmids were precipitated by adding 1:10 (v/v) of 3 M Sodium Acetate (Sigma-Aldrich—no. S2889-250G), followed by four volumes of Absolute Ethanol, and 10 μL of 20 mg/mL Glycogen from oyster (Serve Electrophoresis GmbH—no. 39766.01). Samples were stored for 30 min at—−80°C. Samples were span at 17,000 × *g* for 15 min at 4°C.

DNA pellets were washed three times with 70% ethanol, dried, and resuspended in 30 μL of Alkaline Loading Buffer (300 mM NaOH, 6 mM EDTA, 18% Ficoll, 0.15% Bromocresol Green, and 0.25% Xylene Cyanol FF) and heated to 70°C for 10 min. The alkaline Agarose Gel was prepared by heating Agarose in DEPC-Treated Water (Ambion—no. AM9906) before adding 10 × Alkaline Electrophoresis Buffer (500 mM NaOH, 10 mM EDTA) and GelRed^®^ Nucleic Acid Stain (Biotium—no. 41003) at a 1:10,000 concentration. Samples were loaded and gel was run in denaturing conditions at 30 V for 5 h at 4°C.

## RESULTS

### rAAV quantification and multidimensional ddPCR

Due to vector/template partitioning, ddPCR can be seen as 20,000 independent reactions per sample, thus allowing to analyze rAAVs individually if a single particle is embedded into a droplet. Thus, conditions are highly dependent on the dilution factor used for the absolute quantification. For the latter, Poisson distribution calculates the probability to have one or more particles embedded in a single droplet.

The ability to obtain information for each droplet allows more extensive AAV characterization through multiplexed reactions, whether using the QX ONE (four channels) or the QX 200 (two channels). Our group works with the second one, offering three readouts per droplet, such as channel 1+/channel 2+, channel 1+/channel 2−, and channel 1−/channel 2+, in addition to negative droplets.

The most popular AAV production method is based on plasmid transfection in HEK-293 cells.^16,17^ One drawback is the presence of a low percentage of capsids containing prokaryotic DNA stemming from the plasmid backbone.^12,18,19^ To avoid the *beta-lactamase* gene^20^ regulatory agencies therefore recommend to replace it by a kanamycin resistance gene, or even better, to use prokaryotic sequence-free plasmids.^21^ The pTR-UF11 plasmid used to produce the AAVRSM8 contains the *beta-lactamase* gene in its backbone. Being able to quickly and precisely determine any potential prokaryotic sequences packaged within rAAV particles is essential for thorough characterization.

We have first quantified CMV and *beta-lactamase* gene (beta-lac) as duplex reaction in the AAVRSM8 within the published confidence interval (CI) of the AAVRSM8.^11^

**Figure 1.**
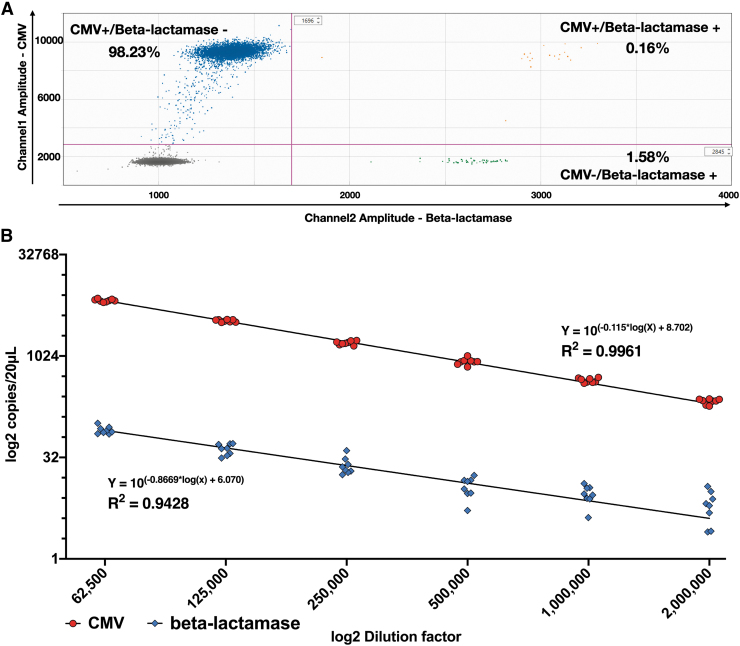
Absolute quantification of CMV and beta-lactamase sequences in the AAVRSM8. **(A)** 2D ddPCR of AAVRSM8 diluted 1,000,000-fold (*n* = 7). *Dot* plot profile of FAM-labeled CMV probe (channel 1) and HEX-labeled beta-lactamase probe (channel 2). Droplets are separated into four groups: channel 1−/channel 2− (*gray dots*), channel 1+/channel 2− (*blue dots*), channel 1−/channel 2+ (*green dots*), and channel 1+/channel 2+ (*orange dots*). The proportion of each group is expressed in percentage of total positive droplets. Differences in single and double populations are delimited by quadrant gates. **(B)** Twofold dilution series of AAVRSM8 analyzed by multiplex ddPCR of CMV and beta-lactamase. Log2 scale is used for X and Y-axes. Each *dot* represents one duplexed ddPCR reaction (*n* = 47). 2D, two dimensions; CMV, cytomegalovirus; FAM, fluorescein; HEX, hexachloro fluorescein; ddPCR, droplet digital PCR.

Our results show that in the AAVRSM8, the beta-lac sequence is present in 1.74% of the AAV particles, distributed in two different droplet populations: CMV+/beta-lac+ and CMV−/beta-lac+ representing 0.16% and 1.58%, respectively (Fig. 1A). The results confirm previously reported results for the AAVRSM8.^12^

As shown above for the two sequences of interest, concentrations can drastically vary within a sample. To obtain robust results, the method linearity for each sequence needs to be monitored. The accurate quantification is highly dependent on the dilution factor and limited by the dynamic range of the ddPCR, thus becoming unreliable when different concentrations between sequences in the same sample is too high. To investigate this linear range, AAVRSM8 was serially diluted in a six-point twofold dilution series (*n* = 8) (between 65,000 and 2,000,000-fold) and ddPCR quantification was performed in a duplex configuration for the simultaneous detection of CMV and beta-lactamase DNA sequences.

CMV titration returns an almost perfect linearity (*R*^2^ = 0.9961) compared with a *R*^2^ = 0.9428 for beta-lac (Fig. 1B). CVs are lower for CMV than for beta-lac (Table 2) at a given dilution. At the highest dilution (2,000,000-fold), the Beta-Lactamase sequence is only detected between 3 and 15 times per reaction through the different replicates, hence leading to a higher CV.

**Table 2.** Evolution of the coefficient of variation of multiplex reactions between cytomegalovirus sequence and beta-lactamase gene from the plasmid backbone

Dilution Factor	CV CMV	CV Beta-Lac
62,500	4.6%	13.3%
125,000	4.1%	18.4%
250,000	6.3%	30.4%
500,000	11.0%	32.7%
1,000,000	7.7%	30.2%
2,000,000	8.9%	50.8%

CV represents the standard deviation divided by the mean expressed in percentage. *n* = 8.

CV, coefficient of variations.

Since titers obtained during a ddPCR experiment result from a statistical calculation based on Poisson distribution, they represent a mean within a CI. We decided to investigate the dispersion of this interval regarding the number of positive droplets and so the dilution factor. To do so, we have added to our linearity assays random dilutions to generate more positive droplets and have analyzed the Poisson CI obtained for both CMV and beta-lac sequences in a duplex configuration.

**Figure 2.**
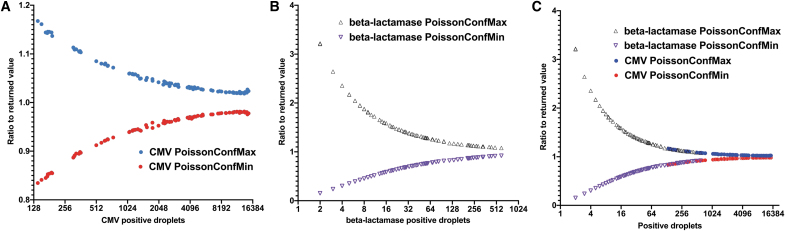
*Dot* plots of Poisson CI in duplexed reaction for CMV and beta-lactamase sequences for the AAVRSM8. Values are presented as a ratio of either the PoissonConfMax (*blue*) or the PoissonConfMin (in *red*) to the returned concentration in relation to the number of positive droplets. **(A)** Ratio to the returned concentration for CMV. *Red dots* represent the PoissonConfMin and *blue dots* the PoissonConfMax. **(B)** Ratio to the returned concentration for beta-lactamase. *Red triangles* represent the PoissonConfMin and *blue triangles* the PoissonConfMax. **(C)** Combination of both data sets (*n* = 96). CI, confidence interval.

As depicted in Fig. 2, the CI is directly linked to the number of positive droplets. The smallest CMV interval (0.98–1.02) was obtained when around 10,000 positive droplets were detected (Fig. 2A) and for beta-lac (0.86–1.14) when as low as 256 positive droplets were detected (Fig. 2B). Nonetheless, the CI returned during the analysis is more scattered at extreme dilutions for beta-lac with a dispersion between 0.1 and 3.2 since only few positive droplets are detected (Fig. 2B).

To perform the most accurate duplex titration possible (with the narrowest Poisson interval for each sequence), it is then necessary to have an optimal number of positive droplets for each sequence, which may be difficult to achieve, especially with AAV preparations of unknown titer.

### Genome integrity

As demonstrated, multidimensional ddPCR allows a more extensive characterization of the packaged DNA compared with qPCR, thanks to the analysis of rAAVs at the single particle level. This is of particular interest for AAV genome integrity^12,14^ since heterogeneity of packaged rAAV genomes has already been described.^22,23^ By duplexing ddPCR reactions using primer/probe sets targeting the promoter (CMV) and the polyadenylation signal (bGH), it is possible to quantify the percentage of rAAV genomes positive for both target sequences simultaneously or either one.

**Figure 3.**
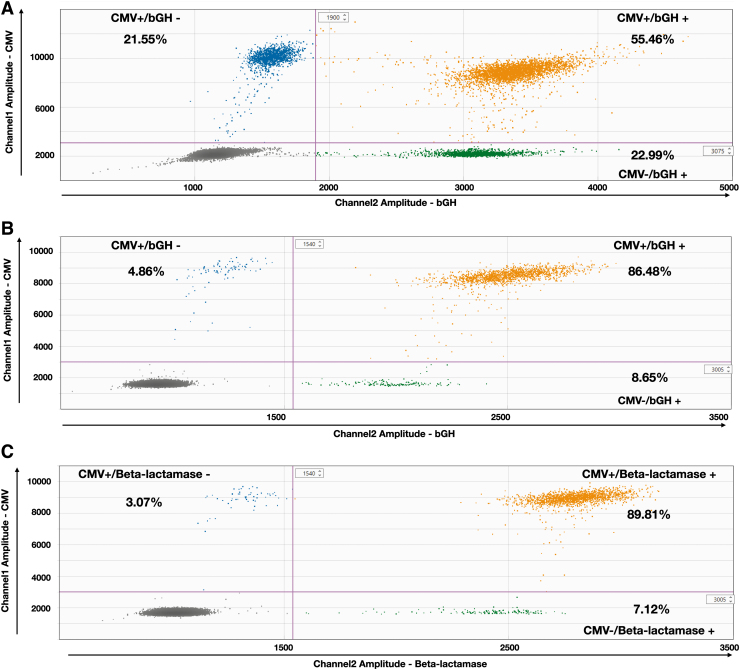
2D ddPCR for genome integrity. *Dot* plot profiles of FAM-labeled channel 1 (CMV) and HEX-labeled channel 2 (bGH or beta-lactamase). Droplets are separated into four groups: channel 1−/channel 2− (*grey dots*), channel 1+/channel 2− (*blue dots*), channel 1−/channel 2+ (*green dots*) and channel 1+/channel 2+ (*orange dots*). The proportion of each group is expressed in percentage of total positive droplets. **(A)** AAVRSM8 for CMV and bGH. **(B)** pTR-UF11 for CMV and bGH. **(C)** pTR-UF11 for CMV and beta-lactamase. bGH, bovine growth hormone.

The ratio between the two values can be interpreted as the “genome integrity”, the closer to 1, the better. As depicted in Fig. 3A, only 55.46% of AAVRSM8 particles are double positive, whereas 21.55% are CMV +/bGH—and 22.99% are CMV -/bGH +. To decipher if this unexpectedly low genome integrity value was due to PCR impeachments at the droplet and/or viral particle level, we performed the ddPCR reaction on a purified pTR-UF11 plasmid batch.

Using plasmid DNA as a template leads to a high percentage of double-positive droplets, either CMV/bGH or CMV/beta-lac, with 86.48% and 89.81% of double-positive droplets, respectively (Fig. 3B, C).

As our group does not have access to the original stock of pTR-UF11 plasmid used to generate the AAVRSM8, we cannot formally exclude that this difference is coming from the quality of the plasmid stock used to generate the reference standard material. To resolve this concern, we have compared the genome integrity values of our rAAV1 vectors produced with our pTR-UF11 plasmid stock. Our rAAV1 batch genome integrity values are similar to those observed for AAVRSM8 (Supplementary Fig. S1A, B), independently of the dilution or positive droplets obtained. The relatively low integrity reported is consistent with the different DNA species observed on a denaturing gel (Supplementary Fig. S1C), which highlights the heterogeneity of packaged rAAV genomes.

The probability to have more than one independent AAV particle in a droplet is corrected during absolute quantification applying the Poisson distribution.^24^ In addition, we were also interested in the correlation between the degree of genome integrity and the dilution factor. Several AAV particles with truncated genomes in a single droplet could indeed affect the genome integrity value (two particles with two different incomplete genomes can result in a double-positive droplet) as previously described.^12^

**Figure 4.**
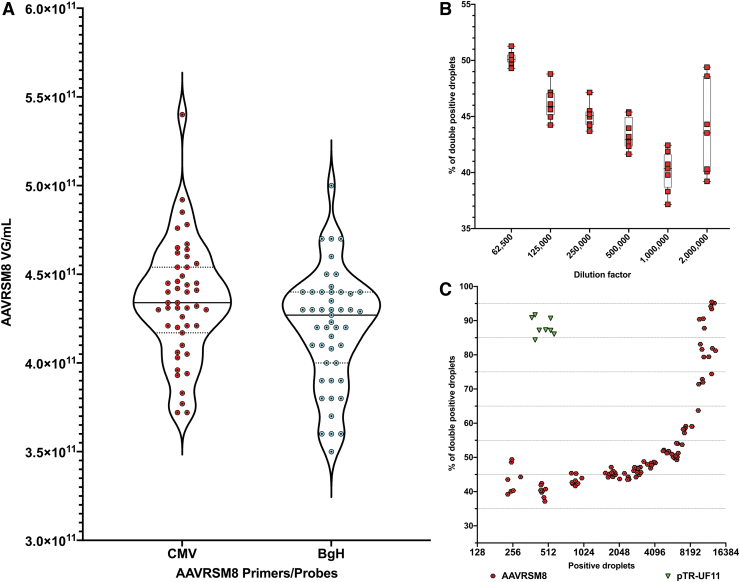
AAVRSM8 CMV and bGH titers and genome integrity by 2D ddPCR. Genome integrity is calculated based on the number of droplets positive for both CMV and bGH compared with the number of total positive droplets and returned as a percentage. **(A)** *Violin* plot of AAVRSM8 vector genome copies per mL for CMV and bGH (*n* = 47). *Plain lines* represent medians, *dotted lines* represent the quartiles. **(B)** *Box* plot of percentage of double-positive droplets for twofold dilution series of AAVRSM8. Eight replicates per dilution. X-axis is represented in log2. *Black lines* represent mean of values. **(C)** Percentage of double-positive droplets correlated to number of total positive droplets for AAVRSM8 (*red circles*) and pTR-UF11 plasmid (*green triangles*). X-axis is represented in log2.

For instance, even with several AAV particles embedded within a droplet, if one of the AAV has a full-length genome, the droplet will return 100% genome integrity, irrespectively of the number of rAAVs in the droplet. On the other hand, where two complementary rAAV genomes are embedded in a single droplet, the quantification error derived from the lower integrity of genomes will be averaged over the number of positive droplets in the well. We repeated our previous twofold dilution experiment by targeting CMV and bGH sequences at the same time. Both titers, CMV and bGH, depicted similar values (Fig. 4A).

At a dilution of 1:62,500, the percentage of double-positive droplets is on average of 50.19%, whereas at a dilution of 1:1,000,000, there are only 40.12% of double-positive droplets. With an additional twofold dilution (1:2,000,000), the average of double-positive droplets is of 43.6% but variability increased between replicates with values ranging from 39.2% to 49.4% (Fig. 4B). At the lowest dilution, AAV particles and positive droplets are in so low amounts per reaction that it increases the variation of the readouts per reaction well.

Since the percentage of droplets positive for both CMV and bGH is directly linked to the number of positive droplets and the probability of several AAV particles per droplet, we investigated if the number of positive droplets correlated with or impacted the percentage of double-positive droplets. Below 4,000 positive droplets, this percentage remains between 40% and 50%. When the number of total positive droplets exceeded this value, the percentage quickly increased to reach 95% at 15,000 droplets (Fig. 4C) as a consequence of multiple AAV particles, containing either partial or full-length genomes, present in one droplet. In contrast, for the pTR-UF11 plasmid batch, this percent integrity value is reached at a much lower concentration, as expected from its demonstrated higher degree of integrity (compare Fig. 4C).

### Impact of primer locations

Assuming that a considerable (±50% for AAVRSM8) proportion of rAAV genomes are not full length, the primer location within the rAAV genome should be absolutely critical. If so, variant primer positioning should lead to substantial variations in quantification, as well as in genome integrity. To investigate the impact of primer positioning, we combined the above-described CMV and bGH primers/probes located at each extremity of the GOI with GFP located in the middle.

Based on our results presented above (Fig. 4B), we decided to analyze AAVRSM8 (*n* = 8) at a dilution of 1.10^−6^ to reliably detect any variation of the genome integrity resulting from several multiplexed reactions (CMV/bGH, CMV/GFP, and GFP/bGH) as well as any titer changes.

**Figure 5.**
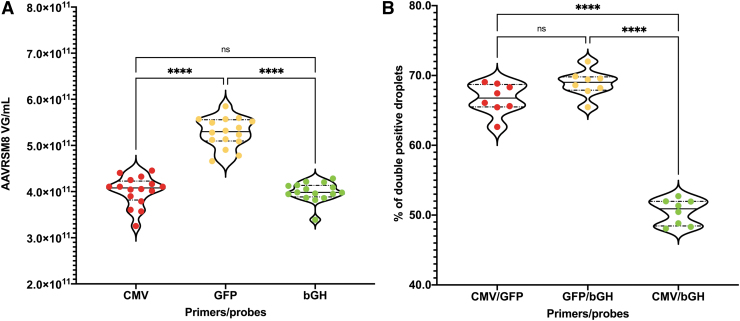
Impact of primer and probe position on AAVRSM8 titration and genome integrity. *Violin* plots of AAVRSM8 ddPCR results regarding three different primers/probe couples used in duplexed reactions. **(A)** AAVRSM8 vector genome copies per mL for each primers/probe pair. CMV—*red dots*, GFP—*yellow dots*, bGH—*green dots* (*n* = 16). **(B)** Percentage of double-positive droplets for three different duplexed primer/probe couples. CMV/GFP—*red dots*, GFP/bGH—*yellow dots*, CMV/bGH—*green dots*. (****p-value <0.0001 following a one-way ANOVA). *Plain black lines* represent the median, and *dotted lines* quartiles. ANOVA, analysis of variance; GFP, green fluorescent protein; ns, not significant.

The respective titers for CMV and bGH returned similar values to those previously described (Fig. 5A). In contrast, the GFP values were considerably higher with an increase of 32% compared with the mean of CMV and bGH values (Fig. 5A). In addition, it appears that genome integrity determination using two-dimensional ddPCR analysis is highly dependent on primer positions. The percentage of droplets positive for both CMV and bGH is only of 50.46% of total positive droplets, whereas it increases to 66.68% and 68.87% for CMV/GFP and GFP/bGH, respectively (Fig. 5B). Droplets only positive for either vector genome extremity (CMV or bGH) represents only a small fraction when duplexed with GFP with 4.24% and 3.84%, respectively (Supplementary Table S1).

This variation of the genome integrity, when either CMV or bGH are duplexed with GFP, is consistent with the increased titer observed with GFP. During rAAV genome replication and packaging, only part of all newly synthesized copies is full length and will return double-positive droplets for CMV and bGH. Some of the genomes will be partially replicated from either ITR and will lead to the packaging of a truncated genome. These truncated or partial rAAV genomes directly affect the value of genome integrity, as well as the rAAV titer depending on the chosen localization of primers and probes.

### Impact of rAAV pretreatment

AAV titration requires many steps, taking place, mostly in plastic tubes. The use of nonionic surfactant such as pluronic acid F-68 to decrease plastic adsorption and to increase titration accuracy has already been described in the ddPCR/qPCR context for several AAV serotypes.^13^ Our group fully agrees with the importance of such addition, also for AAV1 (Supplementary Fig. S2).

The AAV production bulk, before purification, is generally treated with a combination of a lysis agent (or mechanical lysis) and benzonase (or other DNase) to ensure a maximum recovery of intracellular rAAVs and to digest remaining plasmids used for AAV production as well as genomic DNA.^18^ However, before quantification of purified AAV qPCR/ddPCR, an additional DNase treatment is generally recommended to digest any trace of residual nucleic acids.^19,23^

**Figure 6.**
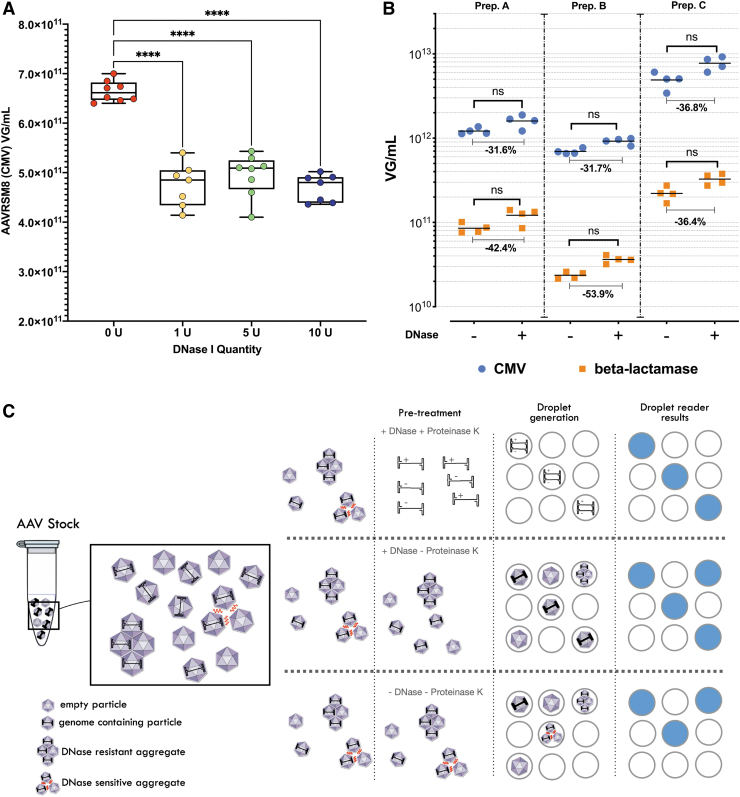
Impact of DNase and proteinase K treatment on ddPCR absolute quantification. **(A)** *Box* plot of AAVRSM8 CMV titer determined by ddPCR. Samples were either pretreated with 1 U (*yellow dots*), 5 U (*green dots*), 10 U (*blue dots*), or without (*red dots*) DNase I before ddPCR titration. No genome extraction was performed (with ****p < 0.0001 following a one-way ANOVA). **(B)** three different rAAV1 batches were either pretreated with 5 U of DNase I before ddPCR titration (+ DNase, *n* = 4) or not (- DNase, *n* = 4). CMV titers are represented in *blue dots*. Beta-lactamase titers are represented in *orange dots*. *Black lines* represent the mean for each quantification. Y-axis in log10 scale. *Gray lines* depict the variations between means. (ns following a two-way ANOVA). Prep. A to C are three different rAAV1 batches produced in our laboratory **(C)**. Three different titration methods are depicted: extracted genomes (+ DNase, + Proteinase K), extraction free with DNase (+ DNase, - Proteinase K), and extraction free (- DNase, - Proteinase K), with the droplet generation profile and the results returned after droplet reader analysis.

We have investigated the necessity and impact of this second DNase pretreatment on purified AAV titration by ddPCR of AAVRSM8 and our own AAV1 batches. We treated the AAVRSM8 with 0.5, 0.25, 0.05 U/μL, or without DNase for 30 min before droplet generation. No titer differences were observed between the different doses of DNase tested, whereas without DNAse pretreatment, ddPCR quantification returned a slightly higher (1.4-fold) CMV concentration (Fig. 6A).

Results upon DNAse treatment observed for our AAV1 batches were surprisingly different from those of AAVRSM8 or from colleagues' previous description.^14^ Indeed, the rAAV1 batches not treated with DNase I depicted a lower titer than the treated samples by at least a 30% decrease when the CMV sequence was targeted, and even higher for beta-lac (between 36% and 53%) (Fig. 6B). This lower titer with untreated samples by DNase cannot be explained by residual plasmid or other nucleic acids, which would have led to the opposite result. Such a pattern was observed with several independently produced AAV1 batches.

Due to the nature of our assay, where AAV capsids are embedded into droplets before ddPCR, we wondered if the observed “decrease” could be explained by the presence of several AAVs in a single droplet. In this case, DNAse treatment could break apart such AAV aggregates and thus increase the number of positive droplets.

AAV particles can aggregate, depending on the serotype, buffer, concentration, *etc.*, and their presence represents an important critical quality attribute as it could impact the transduction efficiency.^25^ AAV aggregation has previously been described as dependent of the ionic strength,^26,27^ and it has been shown that DNase treatment can prevent or at least reduce this phenomenon even at low ionic strength.^26^

Aggregation states also showcase the problematic of genome extraction before ddPCR. Whereas in a qPCR reaction, all rAAV particles (individual particle, DNase sensitive aggregates, or bigger aggregates) are present in the same reaction volume, and in ddPCR, these can be separately embedded into different droplets if rAAV genomes are not extracted before droplet generation. Figure 6C depicts how pretreatment could impact how rAAV genomes or particles are embedded into droplets during a ddPCR assay and how it could impair the droplet reading results.

Treatment with Proteinase K will lead to all rAAV genomes being released (from single particle or any aggregate) and made available for droplet embedding, leading to total rAAV genome quantification (once the strands' reannealing factor 2 is compensated^7^). Whereas, directly embedding AAV particles into droplets could lead to a bias due to several AAV particles being embedded into the same droplet (due to aggregates) and being interpreted as a single AAV particle by the droplet reader and the software.

**Figure 7.**
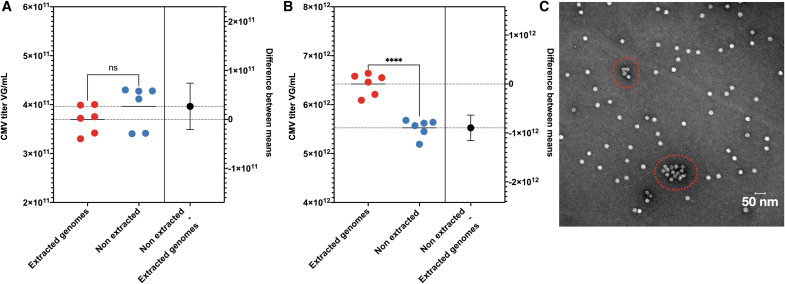
Estimation plot of extracted genomes versus native rAAV titration and TEM image. Estimation plot of CMV ddPCR titration. *Left* Y-axis represents vg/mL, *right* Y-axis difference between means in vg/mL. *Red dots*: quantification of extracted genomes. *Blue dots*: quantification of native AAV particles. Both samples were pretreated with DNase. Mean is represented by *plain and doted lines*. *Black dots* represent the mean of nonextracted genomes + SD by whiskers. *n* = 6; ****p < 0.0001 following an unpaired *t*-test. **(A)** AAV RSM8, **(B)** rAAV1 batch, and **(C)** Negative staining TEM of the rAAV1 batch. Aggregates are highlighted in *red dotted circles*. AAV, adeno-associated virus; rAAV, recombinant AAV; SD, standard deviation; TEM, transmission electron microscopy.

By performing the two “methods” on a single sample, the difference between extracted genomes versus native AAV particles' calculated means could be substantial. Contrary to the AAVRSM8 (Fig. 7A) in one of our samples, the ratio between means for rAAV1 is 1.16 (Fig. 7B) and could provide clues to understand potential AAV states. We have performed electron microscopy (EM) on our AAV preparation and have detected a few aggregates (Fig. 7C). Deciphering if the detected aggregates are responsible for the difference between means is not easy, as we cannot exclude that EM treatment induces AAV particle aggregates.

While EM images allow to identify the quality of an AAV preparation in terms of aggregates, it only represents a small portion of any AAV preparation and should be regarded as additional information. The presence of bigger and/or increased aggregate numbers than those detected in our preparation could lead to an even higher difference in the quantification of extracted and nonextracted genomes by ddPCR.

In summary, our work describes how different results can be obtained in terms of AAV genome quantification and integrity from the same AAV batch depending on dilutions, Poisson distribution, the primers/probes position, and sample treatment before quantification.

## DISCUSSION

The ability to precisely quantify any AAV production is an absolute requirement for an eventual use in clinical trials. Our work describes how the utilization of ddPCR can provide an accurate titer as well as further informations such as packaged prokaryotic sequences, genome integrity and aggregates.

However, as most technics, ddPCR is no exception to potential bias. Limitations due to a lower dynamic range (compared with traditional qPCR) could induce variability in multiplexed reactions when attempting to quantify sequences with important concentration variations. We saw that relatively rare events, such as prokaryotic sequence packaging could be quantified with less confidence than the GOI. This could be even harder with extremely rare events such as replicative competent AAV and/or genomic DNA.^17,19^

Dilution ranges must be carefully determined regarding each rAAV batches to allow optimal linearity with the lowest coefficient of variations and best quantification accuracy. Lower dilutions would likely involve several AAV particles per droplet, whereas higher dilutions will induce higher CVs.

rAAV genome replication is a complex phenomenon starting either from the 5′- or 3′-ITR that appears to potentially fail at different locations within the genome. When attempting to either quantify or characterize rAAV genomes, primers/probes positions should be carefully assessed. If they are located closer to one ITR, both full-length as well as truncated genomes until the position will be detected. On the contrary, by positioning the primers/probes in the middle of the rAAV genome, only full-length genomes will be quantified, as well as truncated ones originating from both ITRs.

Nevertheless, the partitioned reaction allows the individual analysis of each droplet, which in a context of multiplexed reactions allows to estimate the genome integrity of an AAV preparation. Genome integrity can be determined by multiplexing different associations of targets and we have demonstrated that for the AAVRSM8, the different genome integrity values are directly linked to the number of positive droplets as well as the localization of the primer/probe couples. Those two factors also have a direct impact on the returned titer as shown when comparing CMV or bGH to GFP-targeted sequences.

However, despite our efforts, important variations can be detected for rAAV genome integrity when multiplexing ddPCR reactions, since excessively high numbers of positive droplets can lead to an overestimation of the genome integrity values, and excessively low numbers will create higher variability. This major concern should be considered with the greatest care to ensure the most effective quantification and characterization, as difficulties remain in the field.^28^

Furthermore, as described in this article, aggregates could impair rAAV quantification accuracy, as well as potentially affect genome integrity values if several AAV particles are embedded into a single droplet. Orthogonal methods such as long-read sequencing, AUC,^29^ native mass spectrometry,^30^ native and alkaline gel electrophoresis, or more recent charge detection mass spectrometry (CD-MS)^31^ could also provide further insight to determine with even greater accuracy the rAAV genome integrity.

Furthermore, we agree with colleagues^13^ on the absolute necessity to use nonionic surfactant (F-68 pluronic acid for instance) to avoid any plastic adsorption. With this in mind, plastic quality, number of preparation steps before actual titration, and dilution used could affect optimal titration and should be seriously considered.

We were as well interested in how rAAV pretreatment before quantification could potentially impair the titration results. We saw differences between absolute quantification of total vector genomes (cf: extracted genomes) and quantification of droplet-embedded AAV particles with our rAAV1 preparation. An explanation could be the presence of AAV particle aggregates in the analyzed AAV batch. Production and purification processes, serotype used, concentration of samples (dilution used), and buffer compositions could potentially lead to the generation of different kinds of rAAV aggregates, sensitive or not to DNase treatments,^26^ which could potentially affect the quantification values obtained by ddPCR.

As with transmission electron microscopy (TEM), ddPCR analyses only a small fraction of the entire preparation, highlighting the necessity to use other orthogonal methods for a more comprehensive characterization of any rAAV preparation.

From an in-process point of view, we observed differences in the influence of process parameters on final titration. AAVRSM8 and our own rAAV1 preparations indeed behave differently concerning residual nucleic acids. Variations between DNase treated or untreated samples before ddPCR droplet generation could bring further information concerning any residual plasmids or other contaminant as well as the presence of DNase-sensible aggregates. It was previously described that DNA could lead to rAAV aggregates^26^ and this should be taken into consideration before any rAAV quantification by ddPCR.

These three pretreatment options, extraction of total genomes, AAV particles treated or not with DNase could all return different concentrations for the same AAV batch or lot. In our opinion, the non-DNase-treated analysis of AAV particles is closer to the final intended use of the viral preparation. Nonetheless, the closer the three titers, the better as it reflects a more homogeneous rAAV preparation.

To conclude, ddPCR brings more information and accuracy to rAAV quantification and characterization compared with traditional qPCR.^7,9,14^ However, we can only strongly recommend to be extra careful with several parameters, such as (1) dilutions used for absolute quantification, (2) number of positive droplets, (3) primer/probe localization, and (4) pretreatment prior experiment. Sharing all this information could provide deep insight on rAAV batch quality and allow comparative results between groups.

## Supplementary Material

Supplemental data

Supplemental data

Supplemental data
