# Supplementary material for: Insight and Development of Advanced Recombinant Adeno-Associated Virus Analysis Tools Exploiting Single-Particle Quantification by Multidimensional Droplet Digital PCR
Source: Hum Gene Ther. 2022 Sep 16;33(17-18):977–89. doi: 10.1089/hum.2021.182 (PMC10112877; doi:10.1089/hum.2021.182)
Supplement: Supplemental data [file Supp_FigS1.docx]

***Supplementary Figure 1:*** *(A) 2 Dimensions ddPCR for Genome Integrity of GFP rAAV1****.*** *Dot plot profile of FAM-labelled CMV (channel 1) and HEX-labelled bGH (channel 2) n=6. Droplets are separated into 4 groups channel1-/channel2- (grey dots), channel1+/channel2- (blue dots), channel1-/channel2+ (green dots) and channel1+/channel2+ (orange dots). The proportion of each group is expressed in percentage of total positive droplets. Differences in single and double populations are delimited by quadrant gates. (B) Percentage of double positive droplets correlated to number of total positive droplets for AAVRSM8 (red circles) and rAAV1 (blue circles). X axis is represented in log2. (C) Denaturing gel. 1. pTR-UF11 plasmid digested with SmaI endonuclease enzyme, the orange arrow points out the 4171 bp DNA sequence between ITRs. Light purple arrow shows plasmid backbone. 2.* *pTR-UF11 plasmid digested with MscI endonuclease presenting 3 DNA bands (dotted light green arrows) at 3007, 1966 and 1261 bp respectively. 3. rAAV genomes as released from vectors packaged in* *rAAV1 capsids. Orange arrow points out expected full length genome. Truncated or fragmented genomes are presented between doted white brackets.*

**
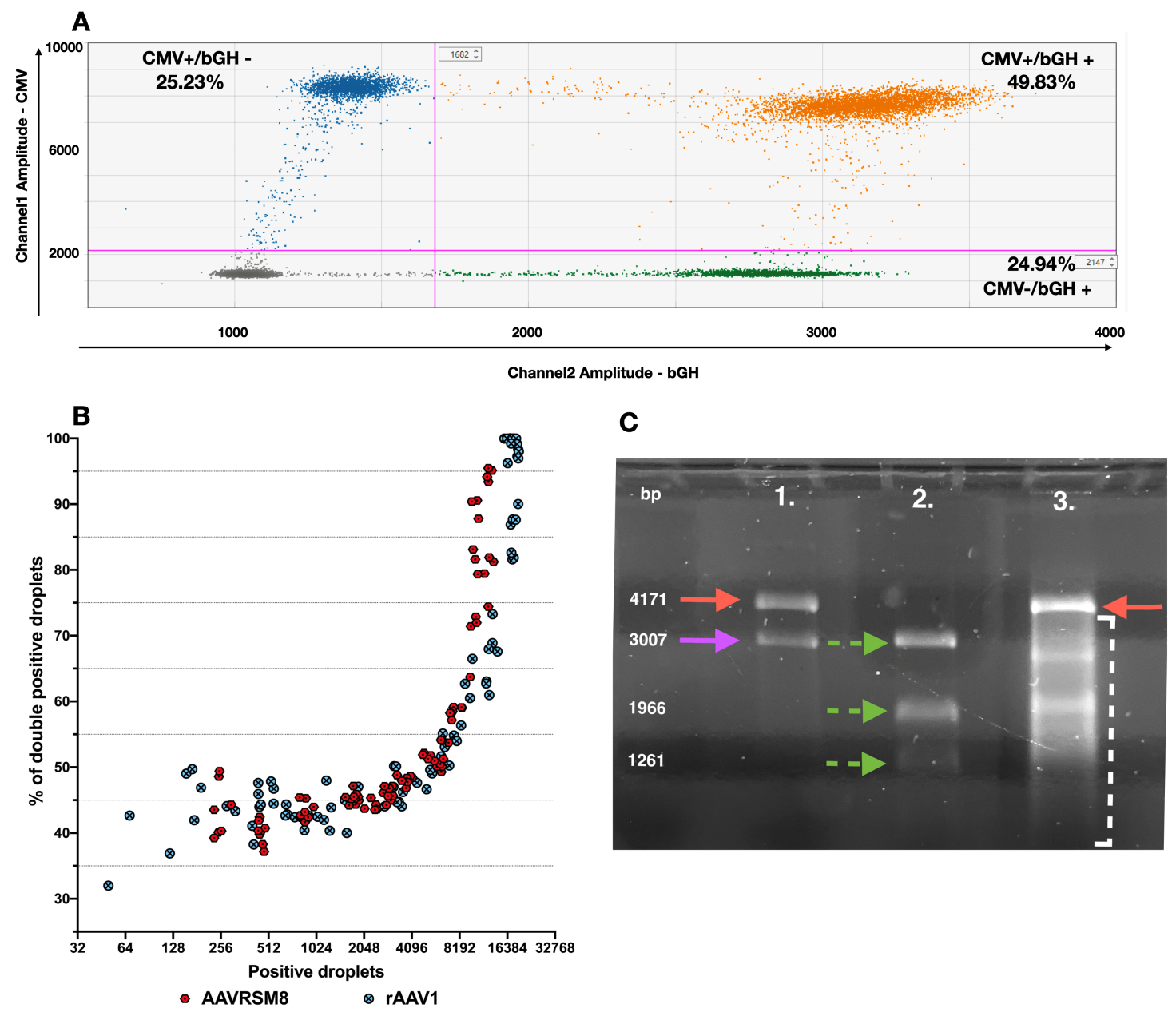
**
