# Supplementary material for: Insight and Development of Advanced Recombinant Adeno-Associated Virus Analysis Tools Exploiting Single-Particle Quantification by Multidimensional Droplet Digital PCR
Source: Hum Gene Ther. 2022 Sep 16;33(17-18):977–89. doi: 10.1089/hum.2021.182 (PMC10112877; doi:10.1089/hum.2021.182)
Supplement: Supplemental data [file Supp_TableS1.docx]

***Supplementary Table 1: Percentage of positive droplets on the AAV RSM8 by 2-dimension multiplex reactions***

| Reaction | CMV only | GFP only | bGH only | CMV+GFP | GFP+bGH | CMV+bGH |
| --- | --- | --- | --- | --- | --- | --- |
| CMV/GFP | **4.24 (0.63)** | **29.07 (2.33)** | **-** | **66.68 (2.17)** | **-** | **-** |
| GFP/bGH | **-** | **27.29 (2.25)** | **3.84 (0.57)** | **-** | **68.87 (1.89)** | **-** |
| CMV/BGH | **25.95 (1.90)** | **-** | **23.59 (1.53)** | **-** | **-** | **50.46 (1.83)** |

*The percentage of positive droplets is calculated by the number of positive droplets for either CMV, GFP, bGH or CMV + GFP, GFP + bGH, CMV+ bGH divided by the total number of positive droplets for each reaction. The values are expressed as the mean percentage of 8 replicates +/- standard deviation.*
