# Supplementary material for: Insight and Development of Advanced Recombinant Adeno-Associated Virus Analysis Tools Exploiting Single-Particle Quantification by Multidimensional Droplet Digital PCR
Source: Hum Gene Ther. 2022 Sep 16;33(17-18):977–89. doi: 10.1089/hum.2021.182 (PMC10112877; doi:10.1089/hum.2021.182)
Supplement: Supplemental data [file Supp_FigS2.docx]

***Supplementary Figure 2: Impact of F-68 Pluronic Acid on CMV ddPCR absolute quantification****. 3 rAAV1 batches were tittered with (blue dots) or without (red dots) 0.05% F-68 pluronic acid during DNase treatment and rAAV particle dilutions, n=6. Black lines represent the mean for each quantification. (*** p-value < 0.001, **** p-value < 0.0001 following a one-way ANOVA). Prep. D to F are three different rAAV1 batches produced in our laboratory*

*
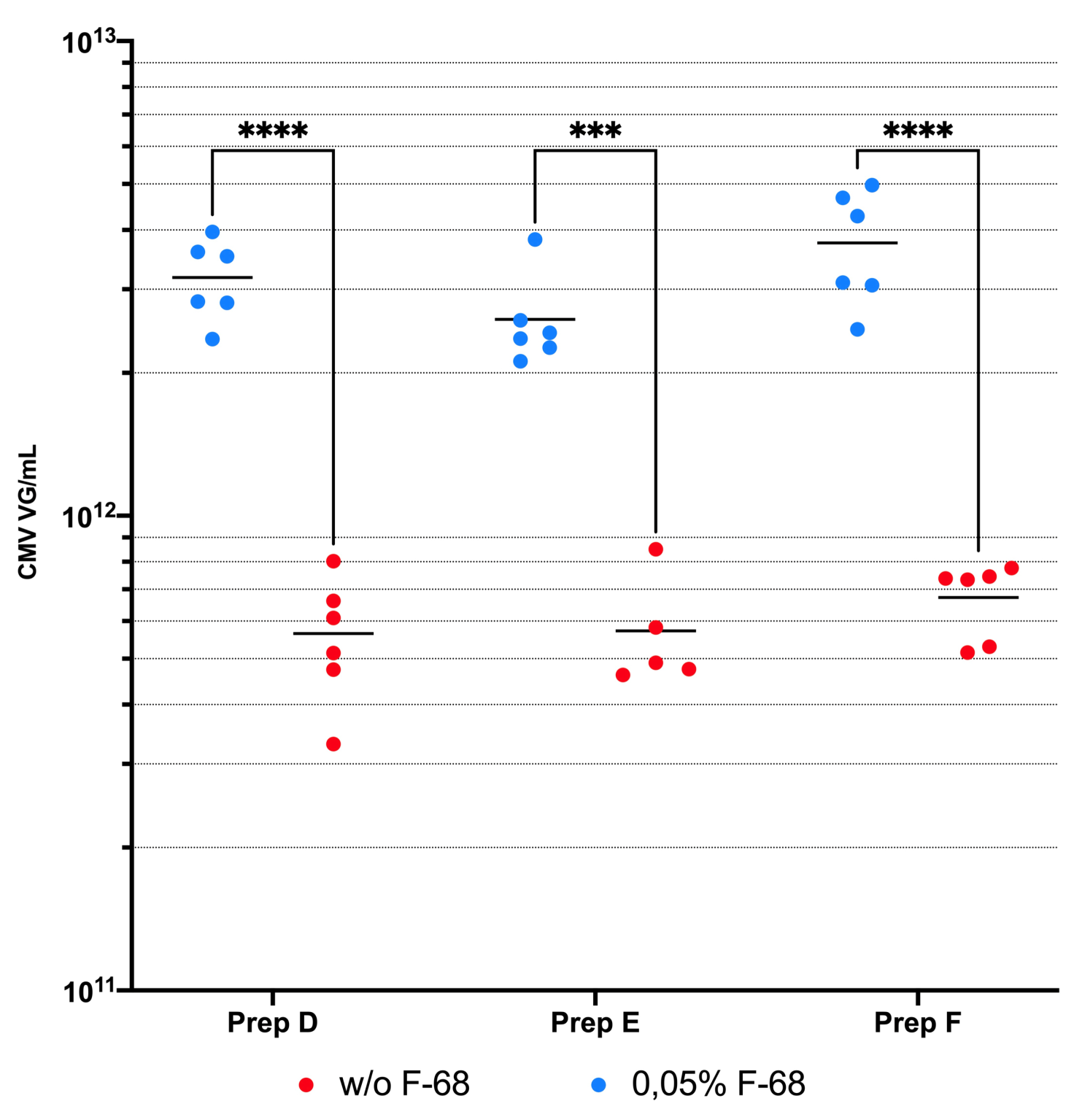
*
